# Genetic structure, relationships and admixture with wild relatives in native pig breeds from Iberia and its islands

**DOI:** 10.1186/1297-9686-45-18

**Published:** 2013-06-14

**Authors:** Luis T Gama, Amparo M Martínez, Inês Carolino, Vincenzo Landi, Juan V Delgado, Antonio A Vicente, José L Vega-Pla, Oscar Cortés, Conceição O Sousa

**Affiliations:** 1 CIISA, Faculdade de Medicina Veterinária, Universidade Técnica de Lisboa, Lisbon, Portugal; 2 Departamento de Genética, Universidad de Córdoba, Campus de Excelencia Internacional Agroalimentario ceiA3, Córdoba, Spain; 3 INIAV, 2005-048, Vale de Santarém, Portugal; 4 Escola Superior Agrária de Santarém, Apartado 310, 2001-904, Santarém, Portugal; 5 Laboratorio de Investigación Aplicada, Cría Caballar de las Fuerzas Armadas, Córdoba, Spain; 6 Departamento de Producción Animal, Universidad Complutense de Madrid, Madrid, Spain

## Abstract

**Background:**

Native pig breeds in the Iberian Peninsula are broadly classified as belonging to either the Celtic or the Mediterranean breed groups, but there are other local populations that do not fit into any of these groups. Most of the native pig breeds in Iberia are in danger of extinction, and the assessment of their genetic diversity and population structure, relationships and possible admixture between breeds, and the appraisal of conservation alternatives are crucial to adopt appropriate management strategies.

**Methods:**

A panel of 24 microsatellite markers was used to genotype 844 animals representing the 17 most important native swine breeds and wild populations existing in Portugal and Spain and various statistical tools were applied to analyze the results.

**Results:**

Genetic diversity was high in the breeds studied, with an overall mean of 13.6 alleles per locus and an average expected heterozygosity of 0.80. Signs of genetic bottlenecks were observed in breeds with a small census size, and population substructure was present in some of the breeds with larger census sizes. Variability among breeds accounted for about 20% of the total genetic diversity, and was explained mostly by differences among the Celtic, Mediterranean and Basque breed groups, rather than by differences between domestic and wild pigs. Breeds clustered closely according to group, and proximity was detected between wild pigs and the Mediterranean cluster of breeds. Most breeds had their own structure and identity, with very little evidence of admixture, except for the Retinto and Entrepelado varieties of the Mediterranean group, which are very similar. Genetic influence of the identified breed clusters extends beyond the specific geographical areas across borders throughout the Iberian Peninsula, with a very sharp transition from one breed group to another. Analysis of conservation priorities confirms that the ranking of a breed for conservation depends on the emphasis placed on its contribution to the between- and within-breed components of genetic diversity.

**Conclusions:**

Native pig breeds in Iberia reveal high levels of genetic diversity, a solid breed structure and a clear organization in well-defined clusters.

## Background

Until the 20th century, pig production throughout Europe was essentially based on local breeds, developed over centuries to fit specific production targets and environmental constraints. The situation changed dramatically with the intensification of agriculture in the mid-20th century, when pig production moved to more intensive systems based on a reduced number of transboundary breeds [1]. This general pattern was largely observed in the Iberian Peninsula, with a unique feature, namely, several outbreaks of African swine fever in the mid 1950’s and 1960’s that resulted in the near disappearance of outdoor pig production and the extinction of the associated native breeds [2]. Reversal of this intensification trend began gradually in the 1980’s, with a renewed interest of consumers for transformed products from local breeds raised in extensive systems. This led producers to attempt to restore the original native pig breeds from the few remaining animals that could be identified. As a consequence, some of these breeds are now well established, but many are still at risk of extinction, given their low census size [See Additional file 1: Table S1].

Native pig breeds in the Iberian Peninsula are broadly classified as belonging to either the Celtic or the Mediterranean breed groups [3]. The Celtic group includes breeds raised in the Northern part of the Iberian Peninsula, characterized by a light skin color, long and bony body, large limbs, brachycephalic head, floppy ears and slow growth rate [4]. The Mediterranean-type pig, often called “Ibérico”, occupies the southern part of the Iberian Peninsula, and is characterized by a dark-colored skin, with a pointed snout, small litters and by the presence of high amounts of subcutaneous and intramuscular fat [5]. Several breeds or varieties are recognized in the Mediterranean group, classified according to their skin and hair color, existence of spots, lack of hair, morphology, etc. [6]. In addition to these two large breed groups, other native pig breeds are raised in more isolated regions, including the Basque region, the Balearic Islands and the Canary Islands, which may or may not have been influenced by the above-mentioned groups. The group classification and census size of the breeds included in our study are summarized in Table S1 [See Additional file 1: Table S1] and the corresponding geographical distribution is illustrated in Figure S1 [See Additional file 2: Figure S1].

A detailed knowledge of the structure and relationships among breeds in a given species is a prerequisite to adopt appropriate conservation strategies and measures [7], which are essential to maintain genetic diversity for the future. Neutral genetic markers, such as microsatellites, are widely used to analyze population structure and relationships and to characterize the genetic diversity of species and populations. In pigs, this approach has been applied to analyze the genetic diversity of several Spanish and Portuguese breeds both at the local level [2,5,8-12], and at a broader level including many European breeds [13,14]. Notwithstanding, some of the more isolated breeds from the Iberian Peninsula were absent from these studies, and it is known that local breeds, many of which are in danger of extinction, contribute significantly to the genetic variability of the species [15]. Today, new statistical tools are available which refine our ability to detect, e.g., how admixture among populations or fragmentation of some of the breeds may have affected their variability and genetic structure. In addition, the existence of wild pigs spread in many areas of the Iberian Peninsula provides the opportunity for admixture with domestic pigs, which has been demonstrated by the existence of wild boar signatures in mitochondrial DNA of Portuguese domestic pigs [16].

In this study, we used a set of microsatellite markers in a comprehensive sample of domestic and wild pig populations from the Iberian Peninsula to: (1) evaluate the existing levels of genetic diversity and corresponding population structure; (2) assess the relationships among breeds and the distinctiveness and homogeneity of the breed groups commonly considered; (3) investigate the possible admixture which may have occurred, both among domestic pig breeds and with their wild relatives; and (4) evaluate conservation alternatives, based on the current levels of between- and within-breed genetic diversity.

## Methods

Individual blood samples were collected from 731 animals representing the major native pig breeds that are recognized in Portugal and Spain, namely the Alentejano (ALE, n = 66), Bísaro (BIS, n = 49) and Malhado de Alcobaça (MAL, n = 36) breeds from Portugal, and the Celta (CEL, n = 27), Chato Murciano (CHM, n = 53), Entrepelado (ENT, n = 73), Euskal Txerria (ETX, n = 56), Lampiño (LAM, n = 59), Manchado de Jabugo (MJA, n = 41), Negro Canario (NCA, n = 53), Negro de Formentera (NFO, n = 21), Negro de los Pedroches (NPE, n = 29), Negro Mallorquín (NMA, n = 20), Retinto (RET, n = 88) and Torbiscal (TOR, n = 60) breeds from Spain. Samples were obtained from animals in different herds and preferably unrelated up to the third generation, in order to capture the largest possible representation of the existing genetic diversity. Blood samples were collected either in slaughter plants or by qualified veterinarians through their routine practice, in the framework of official programs aimed at the identification, health control and parentage confirmation of the breeds and populations included in our study. Therefore, no ethical approval was required for sampling of biological material. In addition to the native pig breeds, 113 samples from wild boars obtained in Spain (SWB, n = 74) and Portugal (PWB, n = 39) were also included in our study. With the exception of the NFO and NPE breeds, all the domestic breeds included in our study are officially recognized and registered in herdbooks. For most breeds, a separate herdbook is kept for each breed, but for the ENT, RET, LAM, MAJ and TOR breeds they are registered as varieties of the “Ibérico” breed, in separate sections of the herdbook. A panel of 24 microsatellite markers was established [See Additional file 1: Table S2], according to the recommendations of FAO and the International Society for Animal Genetics (ISAG) [17]. Allele nomenclature was standardized according to the ISAG Pig Comparison Test, in which we are actively involved. Reference samples are available upon request. Primers were labeled with fluorescent markers to distinguish between fragments of similar size, and microsatellite markers were grouped in multiplex reactions, according to PCR conditions and expected fragment sizes [6]. The PCR products were analyzed by electrophoresis, with an automatic sequencer ABI377XL (Applied Biosystems, Applera Europe B.V.), allele sizing was estimated by using the internal size standard GeneScan-400HD ROX (Applied Biosystems, Madrid, Spain) and a reference sample was also included in each run, to correct for the few variations in allele size assignation among runs. Genotypes were read with the ABI PRISM GeneScan v.3.1.2 software (Applied Biosystems, Madrid, Spain) and interpreted with the ABI PRISM Genotyper v.3.7 NT software (Applied Biosystems, Madrid, Spain).

Different parameters of genetic diversity were estimated and analyzed, as outlined in Table S3 [See Additional file 1: Table S3]. Briefly, the total number of alleles per marker, allele frequencies, observed and expected heterozygosities and effective numbers of alleles per locus were obtained with the Microsatellite toolkit [18] and POPGENE [19], while compliance to Hardy-Weinberg equilibrium was tested using the Fisher’s exact test with the GENEPOP package [20]. Wright’s F-statistics were calculated with the FSTAT package [21] with confidence intervals for the F_IS_ estimates obtained with GENETIX [22], and phylogenetic analyses were carried out with the POPULATIONS software [23], to estimate the genetic distances among breeds. A neighbour-net dendrogram was constructed with the SPLITSTREE4 package [24] based on the matrix of genetic distances, and a tree representing individual genetic distances was obtained with the POPULATIONS software [23]. A hierarchical analysis of variance was performed to partition the total genetic variance into components due to inter-individual, breed or group differences, using the AMOVA (Analysis of Molecular Variance) module of ARLEQUIN 3.5.1.3. [25]. Factors, such as species, country or genetic group, contributing to the genetic variability were assessed, and estimated variance components were used to compute fixation indices. The same software package was used to draw a matrix based on F_ST_ distances among breeds.

The proportion of mixed ancestry in the populations analyzed was evaluated with the Bayesian clustering algorithm implemented by the STRUCTURE v.2.1. computer program [26], which assumes that an individual may have mixed ancestry from different underlying populations and uses multilocus genotypes and a Monte Carlo Markov chain simulation to infer population structure and to assign individuals to the assumed populations. In our case, different numbers of assumed populations (K) were evaluated (from K = 2 to K = 17) with the mixed ancestry model, and the adequateness of the different alternatives was tested by Ln Pr(X|K), i.e., the likelihood of the observed distribution of genotypes given the assumed number of “ancestral” populations. For the different values of K considered, 10 runs of 5*10^5^ iterations were carried out, following a burn-in period of 1*10^5^ iterations, and the results graphically displayed with the DISTRUCT software [27].

Genetic differentiation on a geographical basis was investigated using the R software [28] to draw synthetic contour maps of the Iberian Peninsula, based on the interpolation of genetic contributions to each domestic breed that were computed in the analysis with Structure for K = 3 and considering the center point of the dispersion of each breed.

The relative importance of each domestic breed analyzed for conservation purposes was assessed by considering its contribution to total genetic diversity, assigning different weights to the between- and within-breed components of genetic diversity. First, Weitzman’s approach [29] was applied to calculate the partial contributions of each breed to total genetic diversity, using Reynolds’ genetic distances in the estimation algorithm. Alternatively, the contribution of a breed to within-breed diversity was evaluated by its partial contribution to expected heterozygosity. In an attempt to combine both perspectives, the method of Eding et al. [30] was applied, to analyze the contribution of a breed to a core set using molecular information to estimate the within- and between-breed kinships. After investigating different alternatives to account for molecular coancestries, the weighted log-linear mixed model (WLMM) of Eding and Meuwissen [31] was chosen.

## Results

### Microsatellite markers

For the 24 microsatellite loci analyzed, 304 alleles were detected in the 844 individuals of the 17 populations studied [See Additional file 1: Table S2]. Polymorphism was high for all loci, with the number of alleles per locus ranging from 6 for S0227 to 31 alleles for S0005 and a mean number of alleles per locus of 13.63±3.52. The effective number of alleles and allelic richness accounting for sample size, averaged across loci and breeds, were 3.95±1.93 and 5.41±1.76, respectively. The means across loci for the expected and observed heterozygosities were 0.800±0.062 and 0.718±0.065, respectively, with a within-breed deficit in heterozygosity pooled over loci and breeds of 0.078±0.010. The proportion of genetic variability accounted for by differences among breeds, estimated by *theta* in Table S2 [See Additional file 1: Table S2], was 0.198±0.007 across loci, when all 17 populations were considered. Of the different locus-breed combinations, nearly 20% deviated significantly from Hardy-Weinberg proportions, such that the mean number of breeds showing departure from equilibrium was 3.54±1.84 per locus.

### Breed diversity

Indicators of genetic diversity per breed (Table 1) point toward a higher number of alleles, both in mean number of alleles and allelic richness, in wild populations than in the Iberian domestic breeds. The ETX, MJA, NFO and MAL breeds had the lowest mean number of alleles, effective number of alleles and allelic richness. The ETX, MJA and NFO breeds also had the lowest levels of expected and observed heterozygosity, while wild pigs from Portugal and Spain and the domestic BIS and CEL breeds had the highest levels of genetic diversity. For the whole group of breeds studied, the mean deficit in heterozygosity was 0.078, resulting from either inbreeding, or population substructure (Wahlund effect), or both. Most of the breeds analyzed showed a significant deficit in heterozygosity, which was nearly 0.17 in CEL and NFO, and between 0.1 and 0.15 in CHM, NCA, TOR, SWB and BIS. Most breeds also presented at least one locus not complying with Hardy-Weinberg proportions, with a mean number of 1.65±1.17 loci per breed showing significant deviation from equilibrium.

**Table 1:** Breeds, breed acronyms and breed means and standard deviations for different indicators of genetic diversity

**Breed**	**Acronym**	**MNA**	**NE**	**R**_**t**_	**H**_**e**_	**H**_**o**_	**F**_**IS**_^**a**^	**DHWE**
Celta	CEL	4.54	3.04	4.00	0.596	0.496	0.171	0
		(1.79)	(1.43)	(1.58)	(0.047)	(0.021)	(0.078/0.214)**	
Chato Murciano	CHM	4.58	2.11	3.18	0.464	0.395	0.149	2
		(2.04)	0.71)	(1.12)	(0.044)	(0.014)	(0.089/0.190)**	
Entrepelado	ENT	6.21	2.85	3.84	0.564	0.543	0.036	1
		(2.48)	(1.15)	(1.28)	(0.051)	(0.012)	(−0.007/0.066)	
Euskal Txerria	ETX	4.04	1.95	2.79	0.393	0.389	0.010	2
		(1.99)	(0.84)	(1.21)	(0.052)	(0.013)	(−0.046/0.059)	
Lampiño	LAM	6.08	2.92	4.07	0.573	0.533	0.070	3
		(2.99)	(1.46)	(1.65)	(0.048)	(0.013)	(0.007/0.108)*	
Manchado de Jabugo	MAJ	3.38	1.96	2.75	0.388	0.397	−0.021	1
		(1.64)	(0.94)	(1.23)	(0.051)	(0.016)	(−0.085/0.016)	
Negro Canario	NCA	5.83	2.64	3.87	0.550	0.487	0.116	3
		(1.86)	(1.01)	(1.14)	(0.046)	(0.014)	(0.053/0.160)**	
Negro de Formentera	NFO	3.88	1.91	3.08	0.421	0.351	0.170	1
		(1.62)	(0.67)	(1.11)	(0.044)	(0.022)	( 0.028/0.241)*	
Negro de los Pedroches	NPE	4.21	2.58	3.61	0.561	0.554	0.012	1
		(1.41)	(1.02)	(1.16)	(0.040)	(0.020)	(−0.102/0.082)	
Negro Mallorquín	NMA	4.63	2.51	3.81	0.560	0.514	0.084	0
		(1.47)	(1.01)	(1.11)	(0.034)	(0.023)	(−0.023/0.123)	
Retinto	RET	6.38	2.64	3.83	0.545	0.508	0.068	3
		(2.81)	(1.05)	(1.28)	(0.047)	(0.011)	(0.028/0.097)**	
Torbiscal	TOR	5.00	2.60	3.50	0.527	0.465	0.118	4
		(2.50)	(1.17)	(1.41)	(0.051)	(0.013)	(0.067/0.152)**	
Spanish Wild Boar	SWB	6.50	3.65	4.67	0.592	0.561	0.103	1
		(2.27)	(2.36)	(2.14)	(0.046)	(0.017)	(0.066/0.129)**	
Alentejano	ALE	5.88	3.02	4.07	0.567	0.532	0.063	3
		(2.49)	(1.33)	(1.57)	(0.055)	(0.013)	(0.013/0.096)*	
Bisaro	BIS	5.58	3.34	4.33	0.632	0.549	0.132	1
		(2.26)	(1.56)	(1.44)	(0.040)	(0.015)	(0.071/0.165)**	
Malhado de Alcobaça	MAL	3.67	2.23	3.05	0.522	0.514	0.015	1
		(1.09)	(0.63)	(0.72)	(0.031)	(0.017)	(−0.049/0.050)	
Portuguese Wild Boar	PWB	7.04	3.00	4.44	0.616	0.553	0.055	1
		(3.80)	(1.44)	(1.55)	(0.050)	(0.012)	(−0.010/0.087)	
*Mean*		*5.14*	*2.64*	*3.71*	*0.534*	*0.491*	*0.078*	*1.65*
		*±2.15*	*±1.16*	*±0.57*	*±0.046*	*±0.016*	*(0.060/0.096)*	*±1.17*

^a^Statistical significance of F_IS_ estimate represented as: *P < 0.05 and **P < 0.01; *MNA* number of alleles/locus; *NE* effective number of alleles/locus, *R*_*t*_ allelic richness per locus corrected for breed sample size, *H*_*e*_ expected heterozygosity, *H*_*o*_ observed heterozygosity, *F*_*IS*_ within-population deficit in heterozygosity and confidence interval, *DHWE* number of loci showing deviations from Hardy-Weinberg equilibrium.

### Breed relationships

**Figure 1:**
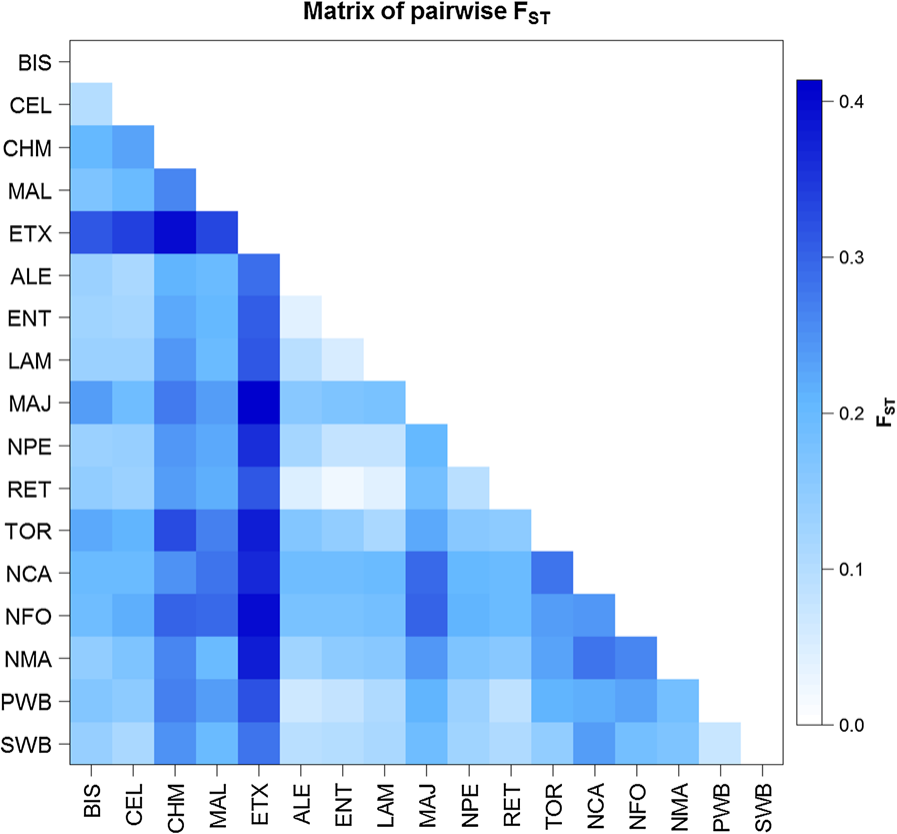
**Graphic representation of the matrix depicting pairwise F**_**ST **_**distances among the 17 pig populations studied.** Colours representing breed distances are defined on the scale at the right side of the figure; breed abbreviations are as defined in Table 1.

The pairwise matrix of F_ST_ distances among the 17 populations studied is shown in Figure 1. Overall, the ETX breed showed the highest degree of differentiation compared to all the other breeds, while the lowest levels of breed differentiation were found among some of the Mediterranean breeds which are considered to be varieties of the “Iberico” pig (RET, ENT, LAM, ALE). The distance between the SWB and PWB populations was also small, but similar to the distance between the PWB and ALE populations.

The Nei D_A_ and Reynolds genetic distances among the 17 populations analyzed are shown in Table S4 [See Additional file 1: Table S4]. The results are similar to those described for the F_ST_ distances, with a stronger differentiation between ETX and the remaining breeds, both in terms of mean distance and individual distances relative to all other breeds. Again, the PWB showed a close proximity to the ALE and RET breeds (D_A_ distance of 0.103 and 0.114, respectively), not much larger than the distance found between PWB and SWB (D_A_ of 0.080).

**Figure 2:**
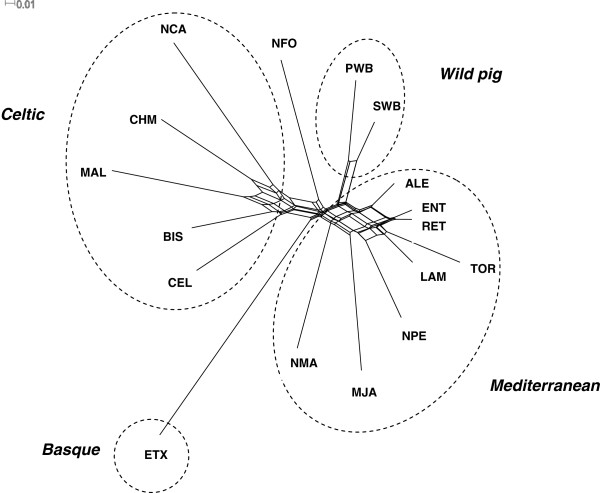
**Neighbour-net dendrogram constructed from D**_**A **_**genetic distances among 15 native pig breeds and two wild populations from Spain and Portugal.** Breed abbreviations are as defined in Table 1.

The neighbour-net drawn from Nei’s D_A_ genetic distances (Figure 2) visualizes the relationships between populations. It identifies three distinct clusters, in addition to two domestic breeds which are clearly separated, i.e., ETX from the Basque region and NFO from the Balearic Islands. The first observed cluster corresponds to the Celtic group of breeds, which includes the CEL, BIS, MAL and CHM breeds, plus the NCA breed from the Canary Islands. Another major cluster includes the Mediterranean-type breeds, i.e., ALE, ENT, RET, TOR, LAM, NPE and MJA, which share a common origin and a close geographical distribution, and are often considered as varieties of the “Iberico” group of pigs. In addition, the NMA breed, which is from the Balearic Islands but has morphological traits similar to the Mediterranean-type group, is also a member of this cluster. The last cluster contains the wild pigs sampled from Portugal and Spain, which are very close to each other and also quite close to the Mediterranean cluster. Indeed, our results suggest a closer proximity between pigs from the Mediterranean cluster and wild boars than Celtic pigs.

**Figure 3:**
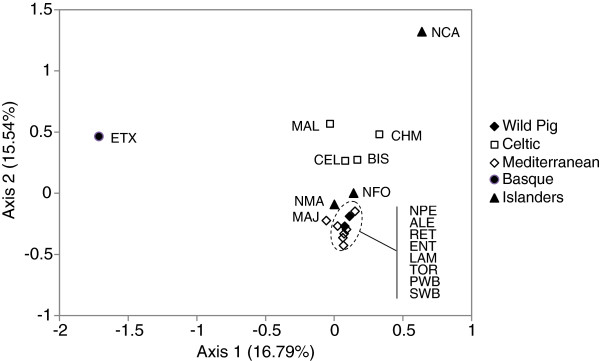
**Spatial representation of genetic distances among the breeds analysed, from the first two axes obtained in the factorial analyses of correspondence.** Values between brackets on both axes represent the contribution in % of each axis to total inertia; breed abbreviations are as defined in Table 1.

In the principal components analysis (PCA), the first three components accounted for nearly 43% of the total variability, and a two-dimension plot (including the first two components) is shown in Figure 3. In this analysis, the ETX and NCA breeds were separated from the other breeds by the first and second axis, respectively. For the remaining breeds, those belonging to the Celtic branch were separated from the Mediterranean branch, while the latter group clustered together with the wild pig populations.

An AMOVA was carried out to investigate the relative contribution of different factors to the observed genetic variability, with each factor considered in a separate analysis, i.e., species (wild vs. domestic pigs), country of origin (Portuguese and Spanish domestic breeds) and genetic group or branch (Celtic, Mediterranean and Basque, considering only the continental Iberian breeds). The results (Table 2) indicate that neither species nor country contribute significantly to genetic differentiation (P > 0.10), while differences among genetic groups accounted for about 12% (P < 0.001) of the observed variability. However, differences among breeds accounted for a significant proportion of the variability (P < 0.001) and were the most important factor in all models.

**Table 2:** Partitioning of genetic variability among different sources of variation by AMOVA, considering the effects of species, country or group

	**Factor considered**		
	**Species**^**a**^	**Country**^**b**^	**Group**^**c**^
Sum of squares / degrees of freedom			
Among factors	193.1 / 1	104.5 / 1	809.7 / 2
Among breeds within factors	2331.4 / 15	2160.8 / 13	949.0 / 9
Within breeds	10380.5 / 1671	8810.6 / 1447	7690.1 / 1262
Variance components			
Among factors	−0.022	−0.181	0.988
Among breeds within factors	1.607	1.742	0.983
Within breeds	6.451	6.319	6.320
Percentage variation			
Among factors	−0.28	−2.30	11.92
Among breeds within factors	20.00	22.11	11.85
Within breeds	80.28	80.19	76.23
F-statistics^d^			
Among factors (F_CT_)	−0.003^*ns*^	−0.023^*ns*^	0.119**
Among breeds within factor (F_SC_)	0.199**	0.216**	0.134**
Among breeds relative to total variability (F_ST_)	0.197**	0.198**	0.119**

^a^wild vs. domestic pigs; ^b^country of origin of domestic breeds: Portuguese vs. Spanish domestic breeds; ^c^groups of continental Iberian domestic breeds, as outlined in Table 1: Mediterranean, Celtic and Basque; ^d^significance of F-statistics: *ns* (P > 0.1) and ** (P < 0.001).

### Population structure

**Figure 4:**
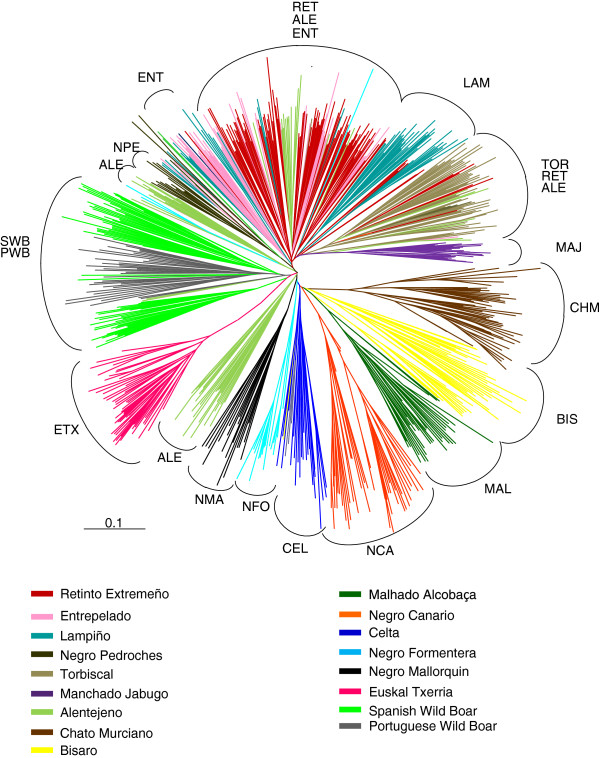
**Radial tree representing neighbour-joining distances among individuals based on allele-sharing (D**_**AS**_**).** Breed abbreviations are as defined in Table 1.

The proportion of shared alleles between animals was used to build a neighbour-joining dendrogram of individuals (Figure 4). The radial tree indicates that, in most cases, animals clustered very well according to breed. However, exceptions to this general pattern are (1) the separation of the SWB animals into two separate clusters that nevertheless clustered with the PWB animals; (2) some of the breeds belonging to the Iberian branch did not completely separate from each other, including for example the RET, ENT and TOR breeds, which overlapped to some degree; and (3) the ALE animals were clustered in several small groups but remained quite homogeneous.

The Bayesian approach implemented by STRUCTURE was used to estimate the most likely number of ancestral populations underlying the observed genetic diversity. The likelihood of the observed data given the number of inferred ancestral populations [Ln Pr(X|K)] is shown in Figure S2 [See Additional file 2: Figure S2], for numbers of inferred populations ranging from K = 2 to K = 17, with ten replications for each value of K. The mean value of Ln Pr(X|K) increased up to K = 16 and then dropped, with a large increase in its variance. Thus, it was assumed that K = 16 is the most likely number of ancestral populations that contribute to the observed genetic variability in the 17 breeds studied.

**Figure 5:**
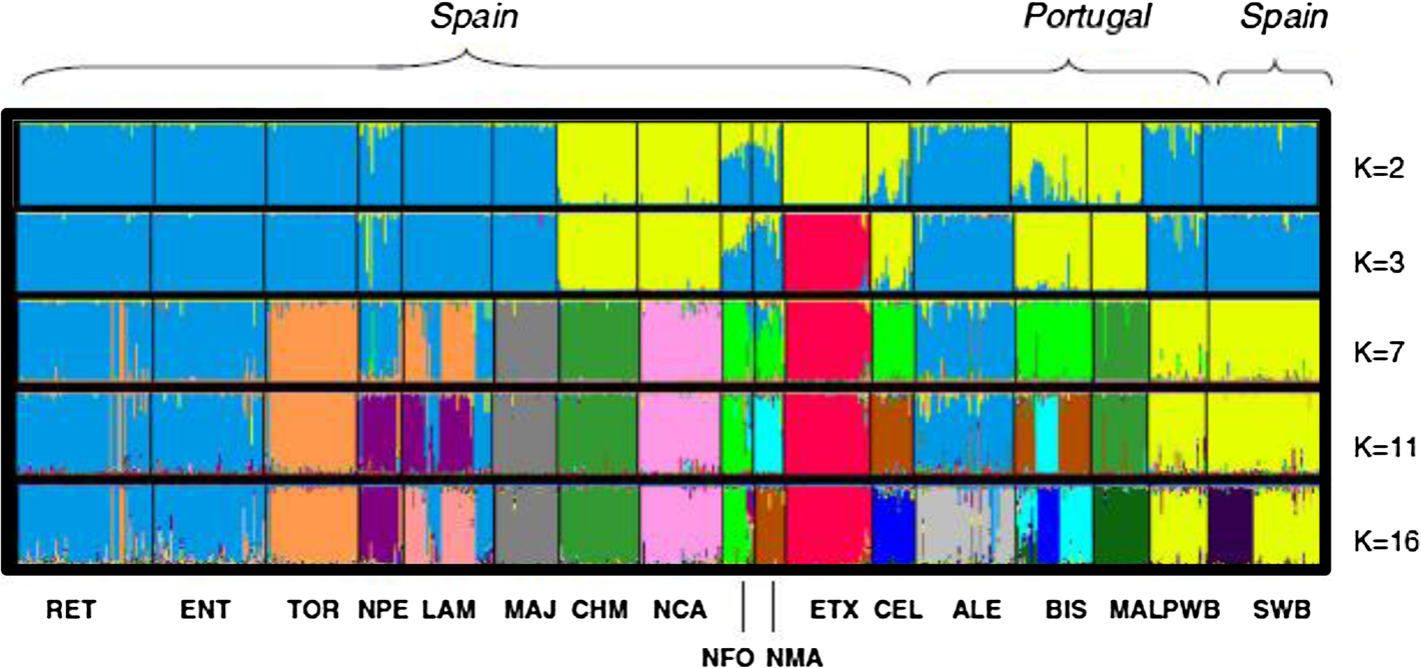
**Population structure of 15 domestic and two wild pig populations inferred by using the STRUCTURE software.** Each animal is represented by a single vertical line divided into K colours, where K is the number of assumed ancestral clusters that ranged from 2 to 16; the colored segment shows the individual’s estimated membership proportions in a given cluster; breed abbreviations are as defined in Table 1.

Contributions of the assumed ancestral populations to each individual of the 17 breeds studied are presented in Figure 5, for values of K ranging between 2 and 16. When K = 2, there is a clear separation between the Celtic breeds and the Mediterranean group and the NMA and NFO breeds have a mixed ancestry. A very relevant feature at K = 2 is that wild pigs clearly share a common ancestry with breeds from the Mediterranean group, but no admixture with the Celtic breeds is detectable. When K = 3, the ETX breed separates completely from the remaining breeds, among which essentially the same relationships are maintained. As K increases, other breeds separate from their original groups and, for example, when K = 7 the wild populations separate from the “Iberico” pigs, the CHM and MAL breeds separate from the remaining Celtic breeds, while the NCA, MJA and TOR breeds separate into independent clusters and ALE, RET, ENT and NPE remain grouped in the same cluster. At this stage, the LAM breed shows some sub-structure, which at K = 11 is stronger and also observed in the BIS breed, while most of the other breeds are associated with a single underlying population. At K = 16, which was estimated as the most likely number of ancestral populations justifying the observed genetic variability in the 17 breeds studied, most breeds are clearly identified with a distinct ancestral population and limited admixture among breeds was detected. Nevertheless, the RET and ENT breeds remain clustered, suggesting that they derive from the same ancestral population. However, there is evidence of a clear sub-structure in some of the breeds studied, including, e.g., SWB, BIS and LAM. All the other breeds are very homogeneous, and associated with a single ancestral population.

### Landscape genetics

**Figure 6:**
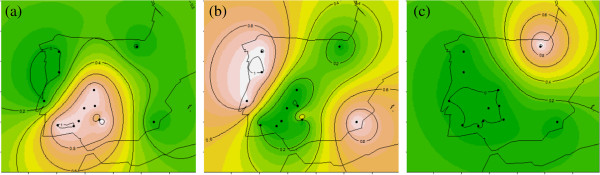
**Synthetic contour maps of the Iberian Peninsula obtained from interpolation of the genetic contributions to each domestic breed estimated in the analysis with STRUCTURE.** The contributions of each of the first three ancestral populations are represented by pink colors, and each sampled breed is represented by a black dot; (**a**) Mediterranean influence; (**b**) Celtic influence; (**c**) Basque influence.

The proportional contributions of the first three inferred ancestral populations to each continental Iberian domestic breed were used to investigate the geographical distribution of genetic differentiation across the Iberian Peninsula (Figure 6). The results of these analyses indicate that the breeds of the Mediterranean group (which are essentially spread in the Southwest of the Iberian Peninsula) differ considerably from those of the Celtic group, and both groups are clearly distinct from the ETX breed which represents the Basque group. Overall, it is also clear that any breed difference that could be due to isolation resulting from national borders was completely overshadowed by genetic differentiation among breed groups, which are spread across borders. However, the distribution observed in Figure 6 mainly reflects the diversity in farm dimension and livestock production systems between the North and Southwest of the Iberian Peninsula, which in turn have a major impact on the type of pig that fits best in a given environment.

### Contribution to genetic diversity

The Weitzman approach prioritizes breeds for conservation based on their contribution to between-breed diversity, and generally more isolated breeds tend to have higher values of partial contributions to genetic diversity. The results of the Weitzman analysis (Table 3) indicate that four breeds showed partial contributions (PC_WTZ_) above 10% (NCA, NFO, ETX and MJA). These correspond to breeds which tend to be more isolated in the neighbour-net of Figure 2 and in the PCA of Figure 3, and which show signs of a strong isolation from the remaining Iberian breeds, i.e., two breeds located in the Canary and Balearic islands (NCA and NFO, respectively), with a reduced census size and a high level of reproductive isolation from Iberian Peninsula breeds, the MJA breed, which is a variety of Iberian pigs, with a very small census size and a narrow geographical distribution in western Andalucía and the ETX breed, which was nearly extinct at the end of the 20th century but has been recovered from a reduced number of animals and is kept relatively isolated in the Basque region.

**Table 3:** **Breed contributions to Weitzman diversity (PC**_**WTZ**_**) and global expected heterozygosity (PC**_**He**_**) and to the core set methodology using the kinship matrix in a weighted log-linear mixed model (WLMM)**

**Breed**	**PC**_**WTZ**_	**PC**_**He**_	**Core WLMM**
CEL	4.7	0.42	8.8
CHM	9.4	−1.76	7.9
ENT	0.9	0.32	6.0
ETX	*15.1*	−1.56	*9.4*
LAM	3.0	0.32	6.7
MAJ	*11.8*	−1.8	0.0
NCA	*10.5*	0.85	*10.9*
NFO	*11.4*	−2	4.2
NMA	6.4	0.66	5.3
NPE	4.9	*1.16*	6.1
RET	1.4	−0.02	4.8
TOR	7.5	−0.12	6.8
ALE	2.7	0.28	5.8
BIS	3.7	*1.58*	*10.7*
MAL	8.6	*1.68*	6.6

Highly prioritised breeds for each case are represented in italic characters.

Another perspective of the contribution to genetic diversity concerns the partial contribution of a breed to expected heterozygosity (PC_He_), which takes into account the within-breed component of genetic variation (Table 1). The highest PC_He_ values were obtained for the MAL, BIS and NPE breeds, and generally the breeds that ranked high in PC_WTZ_ were the ones with the lowest PC_He_. Nevertheless, the NCA and MAL breeds ranked in the top five breeds for both PC_WTZ_ and PC_He_, indicating that they correspond to exceptional situations of high contributions to both within- and between-breed genetic diversity.

Finally, we estimated the contributions of the breeds to a core set, by taking into account their within- and between-breed kinships. In this case, marker-estimated kinships were used in a weighted log-linear mixed model that forces solutions allowing a maximum of one breed with a null contribution to the core set, in addition to weighing marker information to consider the amount of data per locus. Applying the WLMM approach, the NCA, BIS, ETX, CEL and CHM breeds, which have the highest PC_WTZ_ or PC_He_ or at least positive values for both, showed the highest contribution to the core set.

## Discussion

As a result of the variety of environmental conditions, management practices and selection procedures applied during many centuries, a large number of pig breeds have developed over time in the Iberian Peninsula and its archipelagos. These breeds are traditionally classified according to what is considered to be their common origin in two broad groups, i.e., Celtic and Mediterranean [3]. Generally, breeds of the Celtic group are mainly spread in the northern part of the Iberian Peninsula while breeds of the Mediterranean group are mainly located in the southern part. In the past, pigs from any breed of these two groups may have been taken to the Balearic and Canary islands, and have founded or influenced the populations currently existing in those islands. In addition, these populations may have suffered later from the influence of breeds from other origins.

Broadly speaking, pig production systems in the Iberian Peninsula have traditionally followed two very distinct models, which could have a direct impact on the genetic structure of the breeds to which they are associated. These systems roughly follow a geographical pattern. On the one hand, in the northern part of the Iberian Peninsula, where farms tend to be smaller and to have scattered plots, producers usually have a small number of sows, which are traditionally raised in the backyard and fed domestic and horticultural by-products. On the other hand, in the southern regions, farms are much larger and pigs are raised free range and outdoors, where acorn and grass constitute their basic feedstuffs and the ecosystems integrate forest lands which are known as “dehesa” in Spain and “montado” in Portugal [32]. The pigs kept under this system belong to the Mediterranean group and are generally known as “Iberico”, a classification that encompasses several distinct sub-populations which were included in our study.

Our comprehensive analysis of the Iberian pig populations provides important information regarding their genetic diversity and structure, and brings to light new clues on the relationships existing among them and with their wild relatives. The 17 pig populations considered in our study showed considerable levels of genetic diversity, with an overall mean of 13.6 alleles per locus and an average expected heterozygosity of 0.80 for the 24 microsatellite loci considered here. At the breed level, the mean number of alleles per locus was 5.14, with an allelic richness corrected for sample size of 3.71 and an expected heterozygosity of about 0.53. These results are in line with those observed for several European pig breeds, but are somewhat lower than those reported for Asian breeds [33]. The lack of compliance with the Hardy-Weinberg equilibrium that was observed for at least one locus in most breeds is probably related to the overall deficit in heterozygosity in most of the breeds. This deficit could be a consequence of inbreeding or breed substructure, which are common features in local breeds of small census size [13,15], which is the case for nearly all the breeds included here. Moreover, deviations from the Hardy-Weinberg equilibrium due to sampling error are expected given the number of breed-loci combinations and, as expected, these were more often observed in loci with a larger number of alleles [See Additional file 1: Table S2].

On average, genetic diversity was slightly higher in wild pigs than in domestic breeds, possibly as a result of the larger number and wider geographical distribution of the wild pigs. Among the domestic breeds, a few showed strong signs of genetic bottlenecks, expressed as reduced genetic and allelic diversity; this was especially true for breeds with the smallest census size or with a recent history of near-extinction, such as the ETX, MJA, NFO and MAL breeds. Of these breeds, only NFO showed an important deficit in heterozygosity, which was probably a consequence of accumulated inbreeding, while for the other three breeds the F_IS_ estimate was very low or even negative, reflecting the producer’s efforts to favor mating between less related individuals and avoid increased inbreeding in these small populations. The CEL and BIS breeds displayed some common features, including a high allelic richness combined with some of the highest F_IS_ estimates, which may indicate the existence of a fragmented sub-structure in these breeds of the Celtic group, as was also suggested by the analysis with STRUCTURE, especially for the BIS breed. This pattern could result from the fact that these nearly extinct breeds were restored in the 1980’s from a reduced number of animals and then resulted in distinct sub-lines that have been kept separate due to the wide geographical dispersion of the breeds and the very small size and isolation of the herds [34]. However, it has been suggested that exotic genetic material may have been admixed with the BIS breed [35], which could have increased its allelic richness.

In our study, the variability among breeds accounted for about 20% of the total genetic diversity, which is in line with the results reported in other studies on European pig breeds (21%) [13] but is substantially higher than the between-breed diversity reported for other livestock species. The high differentiation among pig breeds reported here could have resulted from the fact that we included both wild and domestic pig populations, and breeds sampled in two different countries. However, this was not the case, since these factors were not significant in the AMOVA, and only the variability among the major domestic breed groups had a significant impact on genetic variability. Indeed, all the different analyses resulted in a clear differentiation between the Celtic, Mediterranean and Basque groups, with all breeds of one group clustering together genetically, and occupying a narrow geographical distribution. The only exception to this local expansion and distribution was the CHM breed, which expanded the northern influence of the Celtic group to the southeastern coast of the Iberian Peninsula.

Consistently, the most distinct feature regarding breed relationships was the separation between the ETX breed and all the other breeds, which could reflect its separate origin and geographical isolation, but is probably also a consequence of its recent re-establishment from a small number of animals. A similar differentiation relative to other breeds was found for the French Basque pig breed [14,36], which is probably related to the ETX breed. A similar pattern of genetic distinctiveness has been reported for Basque human populations, who are considered to be genetic outliers among Europeans [37], confirming the genetic isolation that populations, both human and livestock, have experienced in this region for a long time.

The NFO and NMA breeds from the Balearic Islands did not clearly separate from either the Celtic or the Mediterranean clusters, and the analysis with STRUCTURE confirmed that both could result from the admixture of these two groups. The NCA breed from the Canary Islands seems to share some genetic influences with the Celtic group, but it has become an isolated breed that is very distinct from all the others. Our results could not clearly establish whether this separation resulted from geographical isolation alone or from the possible existence of other influences, since large-scale exchanges are known to have occurred between the Canary Islands and Africa in the past, and perhaps the NCA breed reflects this influence [14]. In any case, the NCA breed was so distinct from all the others that it was consistently ranked in the group with the top conservation priority.

The CEL, BIS and MAL breeds, which are spread in the northwestern part of Iberia, clustered in the neighbour-net with the CHM breed, which is raised mostly in the southeastern coast, forming a well-defined cluster of Celtic breeds. This Celtic group of breeds of the Iberian Peninsula is believed to derive from northern-central European pig breeds [38], with perhaps the introduction of Chinese germplasm in the distant past [39], while the Mediterranean-type pig is assumed to be the pre-extant type in Iberia. One of the most pronounced features in our study was the clear separation between the Celtic and Mediterranean clusters in all the analyses, confirming the large genetic distance and the very distinct breeding practices between the two groups. For example, most of the Celtic breeds had the highest F_IS_ estimates, presumably as a consequence of their very small herd size, which would result in accumulated inbreeding and population fragmentation. Indeed, the BIS breed showed a distinct sub-structure, with two well-defined subpopulations, which may result from the fact that the population was restored in the mid 1980’s from a small number of animals from two distinct regions [2]. The close relationship between the CEL and BIS breeds detected in our study is probably the consequence of their common origin in the recent past [40] while the proximity of the BIS and MAL breeds was already reported in previous studies [2].

As anticipated, no differences could be detected between Spanish and Portuguese wild pigs, but a well differentiated subpopulation was detected in Spain in the analysis with STRUCTURE. This subpopulation corresponds to a genetically isolated group of wild pigs of the National Park of Doñana, kept under highly protective conditions preventing any mixing of the animals with those outside. Furthermore, they probably have a different reproductive behavior, because they are not subject to the increased pressure due to hunting.

The weak differentiation between wild and domestic pig populations found in our study was not completely unexpected, since signs of admixture between these populations have been reported in the past, based on both microsatellite markers [41] and mitochondrial DNA [16]. Indeed, it has been suggested that recurrent backcrossing between domestic animals and their wild relatives is a common event in different species, contributing perhaps to increased genetic diversity [42]. However, in our study with microsatellite markers, the proximity between domestic and wild pigs was only detected in the breeds of the Mediterranean branch, but not in Celtic breeds. This observed admixture could be due to accidental crossbreeding with wild relatives, since in the Mediterranean group, pigs are raised on open range farms throughout the year and fortuitous mating with wild pigs can occur. Such cases are much rarer in Celtic pigs, which are kept in small farms or villages, often in the backyard and in close contact with humans, thus preventing mating with wild pigs. Since wild pigs are not present in the Balearic or Canary Islands, such admixture is virtually impossible in the breeds from these islands.

The different pig breeds of the Mediterranean group sampled in Portugal and Spain clustered together closely (Figures 2 and 3), supporting the existence of a close genetic relationship among what are sometimes referred to as varieties of the “Iberico” pig [6]. Nevertheless, animals from the same breed tended to cluster together (Figure 4) and overall each breed remained with its own structure and identity except for a few cases, including the separation of the ALE breed into four different sub-groups, which clustered with the RET, NPE and TOR breeds or remained isolated. This could result from the existence of distinct sub-lines in the ALE breed that are known to have been admixed with other “Iberico” varieties in the past [43]. In a few cases, some overlapping among varieties of “Iberico” was observed, i.e., between the RET and ENT breeds, which shared a common origin and could not be clearly distinguished from each other, while the LAM breed had a subgroup that also shared a common origin with the RET and ENT breeds. This close relationship among the RET, LAM and ENT breeds probably reflects their past admixture. The TOR, NPE, and MAJ breeds remained isolated from the other “Iberico” varieties, thus confirming their uniqueness and identity.

Overall, the distribution of the Mediterranean influence in pig breeds across the Iberian Peninsula (Figure 6) almost exactly mirrors the distribution of oak and cork forests in the region [44], since these are the basis of the “dehesa-montado” system in which these pigs are traditionally raised. Hence, interdependence between animal and forest resources clearly occurred, which highlights the important role played by native pig breeds towards environmental sustainability.

The definition of conservation priorities for animal genetic resources is an unsolved issue. Different approaches have been proposed to quantify the importance of a given breed for the conservation of genetic diversity [7,29]. However, this should not be the only factor to be taken into account, since livestock breeds have other important features [45,46]. For example, it has been widely recognized that local pig breeds in the Iberian Peninsula are the basis of local high-quality products, and are part of local culture, landscape, traditions, etc., in addition to playing a key role in sustainable development [47].

When conservation strategies focus on the contribution to genetic diversity, priorities will depend on the emphasis given to the between- and within-breed components of genetic diversity, which in turn depends essentially on the choice between a breed that is genetically unique or a breed that has a high genetic diversity, respectively. On the one hand, if only the between-breed contribution is considered (Weitzman approach), the conservation priority will concern the more distinct breeds ETX, MJA, NFO and NCA, which are generally the breeds with the lowest census size, but also have the lowest levels of expected heterozygosity. However, as suggested by Cañon et al. [48], populations with low levels of genetic variability but with distinct features can be combined in crossbreeding strategies, resulting in new populations with high levels of heterozygosity. On the other hand, if the within-breed component of genetic diversity (heterozygosity approach) is considered, the conservation priority will concern the MAL, BIS, NPE and NCA breeds. This is the consequence of the high heterozygosity level of these breeds. However, in this case, three of the four breeds chosen for conservation belong to the Celtic group, which could be difficult to accept from a practical point of view.

A more balanced solution would be to assess conservation priorities on the basis of the contribution of each breed to the overall diversity, based on its molecular contribution to between- and within-breed kinships. In our case, the priority would be given to the NCA, BIS, ETX and CEL breeds, which contribute most to the between- or within-breed components of genetic diversity. Thus, this solution represents a compromise between the two previous methods but no breed from the Mediterranean group would be selected for conservation, which would be in essence difficult to justify.

Overall, our study of conservation priorities confirms that there is no single recommended approach, and that, depending on the emphasis placed on the between- or within-breed components of genetic diversity, the ranking of breeds for conservation may be very different, as discussed by Cañon et al. [48]. Furthermore, when the contribution of each breed is considered individually, there is a risk that some clusters of breeds will be completely left out of the conservation priorities, which may not be desirable in practice. It is therefore important to further elaborate on the priorities of conservation of animal genetic resources, to account not only for breed contributions towards genetic variability, but also for their production and adaptation features, as well as for their economic, demographic, social, ecological, and cultural importance.

The analysis of genetic diversity and conservation priorities based on neutral genetic markers, as carried out in our study, does not take into account the genetic variability of markers associated with production and adaptation traits, which often differ considerably among breeds. In recent years, high-density panels were developed capable of simultaneously detecting variability in thousands of single nucleotide polymorphisms (SNP), and these could be instrumental in detecting genomic regions associated with both neutral and non-neutral sources of genetic variability. Hence, these SNP chips will provide a deeper insight into genetic diversity within- and between-breeds, population structure and selection signatures and their use in genetic diversity studies is very promising [49].

## Conclusions

Our results indicate that the genetic diversity in native pig breeds of the Iberian Peninsula is high and that the local isolated breeds, often with a small census size, are important reservoirs of genetic diversity. Several breeds had a significant deficit in heterozygosity, probably as a consequence of accumulated inbreeding, and the MJA and MAL breeds showed signs of genetic erosion. Nearly 20% of the observed diversity is due to differences among breeds, which is mostly due to variability among the Celtic, Mediterranean and Basque groups that are organized in very distinct breed clusters. The genetic distance between wild and domestic pig populations is small, and clear signs of admixture are observed between breeds from the Mediterranean group and their wild relatives but less so in the Celtic group. The Mediterranean populations included in our study represented varieties of the so-called “Iberico” pig group but, in spite of their close distribution, most of the varieties of this group were genetically distinct from each other and well-structured, while a few showed signs of admixture and/or fragmentation. The results presented here are useful to define conservation priorities and to adopt management strategies aimed at minimizing further losses of genetic diversity in the future.

## Competing interests

The authors declare that they have no competing interests.

## Authors’ contributions

AMM, JVD, LTG conceived and designed the experiments; AAV, AMM, COS, IC, VL performed the experiments; AMM, JLV, LTG, OC, VL analyzed the data; AMM, JVD, LTG wrote the manuscript; AAV, COS, JLV, IC, OC, VL reviewed and edited the manuscript; Members of the BIOPIG Consortium provided biological samples and logistic support. All authors read and approved the final manuscript.

## Supplementary Material

Additional file 1: Table S1 Breeds studied, acronyms, breed groups, approximate census size and sample size used in the present study. Baseline information for the breeds included in the study. **Table S2**. Genetic diversity parameters by locus across 17 pig populations. Total and effective number of alleles, allelic richness, heterozygosities and F-statistics per locus. **Table S3**. List of software used for statistical analyses. Programs used and corresponding parameters estimated. **Table S4**. Nei’s D_A_ and Reynolds genetic distances among 17 Iberian pig populations. Pairwise genetic distances among the breeds studied. Click here for file

Additional file 2: Figure S1 Geographic distribution of the domestic pig breeds studied. Map showing spread of the 15 domestic breeds analysed. **Figure S2**. Estimated posterior probabilities of the data for different number of inferred clusters in the analysis with Structure. Likelihood of different number of ancestral populations given the observed breed genetic diversity. Click here for file
