# Supplementary material for: Genetic structure, relationships and admixture with wild relatives in native pig breeds from Iberia and its islands
Source: Genet Sel Evol. 2013 Jun 14;45(1):18. doi: 10.1186/1297-9686-45-18 (PMC3698160; doi:10.1186/1297-9686-45-18)
Supplement: Additional file 1: Table S1 — Breeds studied, acronyms, breed groups, approximate census size and sample size used in the present study. Baseline information for the breeds included in the study. Table S2. Genetic diversity parameters by locus across 17 pig populations. Total and effective number of alleles, allelic richness, heterozygosities and F-statistics per locus. Table S3. List of software used for statistical analyses. Programs used and corresponding parameters estimated. Table S4. Nei’s DA and Reynolds genetic distances among 17 Iberian pig populations. Pairwise genetic distances among the breeds studied. [file 1297-9686-45-18-S1.docx]

Table S1. Breeds studied, acronyms, breed groups, approximate census size and sample size used in the present study.

| Breed | Acronym | Breed Group | Census size^a^ | Sample size |
| --- | --- | --- | --- | --- |
| Celta | CEL | Celtic | 780 | 27 |
| Chato Murciano | CHM | Celtic | 290 | 53 |
| Entrepelado | ENT | Mediterranean | 19237 | 73 |
| Euskal Txerria | ETX | Basque | 300 | 56 |
| Lampiño | LAM | Mediterranean | 951 | 59 |
| Manchado de Jabugo | MAJ | Mediterranean | <100 | 41 |
| Negro Canario | NCA | Islanders | 344 | 53 |
| Negro de Formentera | NFO | Islanders | <100 | 21 |
| Negro de los Pedroches | NPE | Mediterranean | <100 | 29 |
| Negro Mallorquín | NMA | Islanders | 1126 | 20 |
| Retinto | RET | Mediterranean | 72299 | 88 |
| Torbiscal | TOR | Mediterranean | 1600 | 60 |
| Spanish Wild Boar | SWB | Wild | - | 74 |
| Alentejano | ALE | Mediterranean | 6525 | 66 |
| Bisaro | BIS | Celtic | 2521 | 49 |
| Malhado de Alcobaça | MAL | Celtic | 229 | 36 |
| Portuguese Wild Boar | PWB | Wild | - | 39 |

^a^Estimated census sizes for Spanish breeds were obtained from ARCA at http://aplicaciones.magrama.es/arca-webapp/flujos.html?_flowId=catalogoRazas-flow&_flowExecutionKey=e4s1#, and for Portuguese breeds from DGAV at [http://www.dgv.min-agricultura.pt/xeov21/attachfileu.jsp?look_parentBoui= 3820310&att_display=n&att_download=y](http://www.dgv.min-agricultura.pt/xeov21/attachfileu.jsp?look_parentBoui=%203820310&att_display=n&att_download=y%20) both consulted on September 30th, 2012. No reliable estimated population data exists for wild boars, but census sizes are believed to exceed 40 000 in Portugal and 150 000 in Spain.

Table S2. Microsatellite loci analysed and corresponding genetic diversity indicators estimated across 17 pig populations^a^.

| **Locus** | **NA** | **NE** | **R_t_** | **H_e_** | **H_o_** | ***f*** | *theta* | **HWE** |
| --- | --- | --- | --- | --- | --- | --- | --- | --- |
| *IGF1* | 11 | 4.33 | 5.56 | 0.769 | 0.602 | 0.088±0.041 | 0.147±0.033 | 6 |
| *S0002* | 15 | 4.66 | 6.20 | 0.786 | 0.542 | 0.132±0.027 | 0.215±0.048 | 3 |
| *S0005* | 31 | 7.06 | 9.22 | 0.859 | 0.552 | 0.189±0.039 | 0.217±0.042 | 8 |
| *S0026* | 12 | 2.30 | 4.52 | 0.566 | 0.400 | 0.142±0.052 | 0.188±0.040 | 6 |
| *S0068* | 20 | 8.50 | 8.74 | 0.883 | 0.673 | 0.068±0.037 | 0.190±0.045 | 6 |
| *S0090* | 8 | 3.65 | 4.73 | 0.726 | 0.557 | 0.084±0.022 | 0.176±0.079 | 3 |
| *S0101* | 13 | 3.77 | 6.12 | 0.735 | 0.513 | 0.081±0.030 | 0.251±0.052 | 2 |
| *S0155* | 12 | 3.03 | 4.08 | 0.670 | 0.490 | 0.084±0.039 | 0.212±0.076 | 2 |
| *S0178* | 12 | 7.49 | 7.95 | 0.867 | 0.642 | 0.094±0.042 | 0.191±0.028 | 5 |
| *S0215* | 8 | 1.28 | 2.44 | 0.221 | 0.170 | 0.080±0.067 | 0.183±0.050 | 2 |
| *S0225* | 11 | 2.29 | 4.59 | 0.564 | 0.452 | 0.027±0.039 | 0.187±0.059 | 3 |
| *S0226* | 16 | 3.60 | 4.99 | 0.723 | 0.534 | 0.076±0.036 | 0.204±0.059 | 2 |
| *S0227* | 6 | 1.25 | 2.53 | 0.202 | 0.161 | 0.054±0.064 | 0.166±0.060 | 1 |
| *S0228* | 13 | 2.27 | 5.01 | 0.559 | 0.465 | -0.033±0.057 | 0.211±0.060 | 2 |
| *S0355* | 11 | 1.34 | 2.99 | 0.255 | 0.124 | 0.254±0.143 | 0.399±0.121 | 1 |
| *S0386* | 14 | 4.54 | 6.30 | 0.780 | 0.592 | 0.082±0.040 | 0.181±0.046 | 4 |
| *SW024* | 15 | 4.42 | 5.89 | 0.774 | 0.572 | 0.022±0.031 | 0.259±0.069 | 2 |
| *SW072* | 16 | 4.13 | 5.74 | 0.758 | 0.596 | 0.060±0.025 | 0.175±0.055 | 4 |
| *SW240* | 11 | 4.51 | 5.36 | 0.779 | 0.631 | 0.057±0.029 | 0.147±0.031 | 4 |
| *SW632* | 11 | 4.86 | 6.73 | 0.795 | 0.600 | 0.051±0.031 | 0.217±0.057 | 6 |
| *SW857* | 9 | 4.99 | 5.73 | 0.800 | 0.623 | 0.027±0.032 | 0.209±0.063 | 4 |
| *SW911* | 8 | 3.85 | 5.10 | 0.741 | 0.587 | 0.057±0.032 | 0.167±0.031 | 3 |
| *SW936* | 12 | 5.25 | 6.53 | 0.810 | 0.605 | 0.107±0.034 | 0.174±0.043 | 4 |
| *SW951* | 9 | 1.36 | 2.84 | 0.264 | 0.223 | 0.021±0.071 | 0.148±0.026 | 2 |
| *Means(SD)* | *13.63±3.52* | *3.95±1.93* | *5.41±1.76* | *0.800±0.062* | *0.718±0.065* | *0.078±0.010* | *0.198±0.007* | *3.54±1.84* |

^a^ Total number of alleles (NA), effective number of alleles (NE), mean allelic richness per locus corrected for breed sample size (R_t_), expected (H_e_), and observed (H_o_) heterozygosities, and F-statistics *f* (amount of inbreeding within populations) and *theta* (amount of differentiation among populations), and number of breeds showing deviations from Hardy-Weinberg equilibrium (HWE) in each locus (p < 0.05)

Table S3. List of software used for statistical analyses

| **Program** | **URL** | **Reference** | **Parameters calculated** |
| --- | --- | --- | --- |
| **MICROSATELLITE TOOLKIT** | <http://www.animalgenomics.ucd.ie/sdepark/ms-toolkit/> | [18] | Allele frequencies, total number of alleles per locus, estimated observed (Ho) and unbiased expected (He) heterozygosities per locus  Mean number of alleles, observed and unbiased expected estimates of heterozygosity per population and their standard deviations |
| **POPGENE** | <http://www.ualberta.ca/~fyeh/index.htm> | [19] | Effective number of alleles |
| **GENEPOP** | <http://kimura.univ-montp2.fr/~rousset/Genepop.htm> | [20] | Deviations from Hardy–Weinberg equilibrium |
| **FSTAT** | [http://www2.unil.ch/popgen/softwares/fstat.htm](http://www2.unil.ch/popgen/) | [21] | F-statistics *f* and *theta* per locus (Weir & Cockerham 1984) and the corresponding P-values obtained based on 1000 randomizations  Allelic richness over all loci per breed |
| **GENETIX** | http://kimura.univ-montp2.fr/genetix/ | [22] | F_IS_ with a 95% confidence interval, determined by 1000 permutations and 10 000 bootstraps across loci |
| **POPULATIONS** | <http://bioinformatics.org/~tryphon/populations/> | [23] | Genetic distances |
| **SPLITSTREE4** | <http://www-ab.informatik.uni-tuebingen.de/software/splitstree4> | [24] | Construction of a neighbour-net dendrogram |
| **ARLEQUIN** | <http://cmpg.unibe.ch/software/arlequin35/> | [25] | AMOVA (Analysis of Molecular Variance) |
| **STRUCTURE** | <http://pritch.bsd.uchicago.edu/structure.html> | [26] | Investigation of the genetic structure of the populations |
| **DISTRUCT** | <http://rosenberglab.bioinformatics.med.umich.edu/distruct.html> | [27] | Used to graphically display results produced by the genetic clustering program [STRUCTURE](http://pritch.bsd.uchicago.edu) |
| **R** | http://www.r-project.org/ | [28] | Drawing of synthetic contour maps representing interpolated genetic contributions of ancestral populations to each domestic breed |

Table S4. Pairwise D_A_ (above diagonal) and Reynolds (below diagonal) genetic distances among 17 Iberian pig populations

|  | **CEL** | **CHM** | **ENT** | **ETX** | **LAM** | **MAJ** | **NCA** | **NFO** | **NPE** | **NMA** | **RET** | **TOR** | **SWB** | **ALE** | **BIS** | **MAL** | **PWB** | **Mean** |
| --- | --- | --- | --- | --- | --- | --- | --- | --- | --- | --- | --- | --- | --- | --- | --- | --- | --- | --- |
| Celta | - | 0.299 | 0.259 | 0.430 | 0.276 | 0.308 | 0.331 | 0.323 | 0.276 | 0.306 | 0.253 | 0.325 | 0.248 | 0.241 | 0.209 | 0.291 | 0.288 | 0.291 |
| Chato Murciano | 0.256 | - | 0.302 | 0.481 | 0.315 | 0.337 | 0.315 | 0.334 | 0.322 | 0.332 | 0.312 | 0.375 | 0.326 | 0.286 | 0.278 | 0.292 | 0.351 | 0.329 |
| Entrepelado | 0.147 | 0.258 | - | 0.395 | 0.093 | 0.205 | 0.341 | 0.258 | 0.153 | 0.230 | 0.043 | 0.145 | 0.170 | 0.082 | 0.236 | 0.352 | 0.212 | 0.217 |
| Euskal Txerria | 0.422 | 0.532 | 0.356 | - | 0.401 | 0.431 | 0.506 | 0.450 | 0.432 | 0.421 | 0.389 | 0.429 | 0.403 | 0.366 | 0.436 | 0.430 | 0.427 | 0.427 |
| Lampiño | 0.158 | 0.275 | 0.064 | 0.378 | - | 0.233 | 0.332 | 0.290 | 0.148 | 0.225 | 0.091 | 0.127 | 0.186 | 0.135 | 0.263 | 0.351 | 0.218 | 0.230 |
| Manchado de Jabugo | 0.274 | 0.381 | 0.182 | 0.521 | 0.229 | - | 0.444 | 0.328 | 0.266 | 0.298 | 0.198 | 0.234 | 0.305 | 0.205 | 0.363 | 0.388 | 0.335 | 0.305 |
| Negro Canario | 0.253 | 0.331 | 0.271 | 0.503 | 0.264 | 0.457 | - | 0.383 | 0.352 | 0.407 | 0.356 | 0.403 | 0.365 | 0.360 | 0.322 | 0.386 | 0.366 | 0.373 |
| Negro de Formentera | 0.271 | 0.369 | 0.223 | 0.573 | 0.247 | 0.408 | 0.367 | - | 0.300 | 0.317 | 0.249 | 0.285 | 0.297 | 0.240 | 0.285 | 0.409 | 0.312 | 0.316 |
| Negro de los Pedroches | 0.151 | 0.277 | 0.095 | 0.438 | 0.090 | 0.253 | 0.277 | 0.251 | - | 0.293 | 0.157 | 0.192 | 0.257 | 0.211 | 0.269 | 0.356 | 0.285 | 0.267 |
| Negro Mallorqín | 0.182 | 0.299 | 0.149 | 0.439 | 0.148 | 0.297 | 0.319 | 0.321 | 0.187 | - | 0.213 | 0.279 | 0.255 | 0.187 | 0.286 | 0.358 | 0.276 | 0.293 |
| Retinto | 0.169 | 0.286 | 0.026 | 0.374 | 0.066 | 0.183 | 0.291 | 0.241 | 0.118 | 0.164 | - | 0.130 | 0.177 | 0.084 | 0.244 | 0.362 | 0.213 | 0.217 |
| Torbiscal | 0.228 | 0.358 | 0.138 | 0.452 | 0.118 | 0.265 | 0.340 | 0.273 | 0.157 | 0.230 | 0.145 | - | 0.215 | 0.164 | 0.321 | 0.424 | 0.284 | 0.271 |
| Spanish Wild Boar | 0.128 | 0.264 | 0.110 | 0.336 | 0.111 | 0.251 | 0.284 | 0.240 | 0.148 | 0.171 | 0.129 | 0.143 | - | 0.153 | 0.241 | 0.355 | 0.136 | 0.256 |
| Alentejano | 0.137 | 0.231 | 0.039 | 0.329 | 0.086 | 0.173 | 0.275 | 0.213 | 0.123 | 0.121 | 0.053 | 0.152 | 0.100 | - | 0.230 | 0.336 | 0.191 | 0.217 |
| Bisaro | 0.110 | 0.219 | 0.131 | 0.371 | 0.139 | 0.294 | 0.234 | 0.231 | 0.140 | 0.146 | 0.155 | 0.214 | 0.131 | 0.136 | - | 0.269 | 0.280 | 0.283 |
| Malhado de Alcobaça | 0.209 | 0.296 | 0.249 | 0.442 | 0.231 | 0.374 | 0.337 | 0.389 | 0.257 | 0.222 | 0.268 | 0.321 | 0.241 | 0.236 | 0.192 | - | 0.367 | 0.358 |
| Portuguese Wild Boar | 0.163 | 0.295 | 0.114 | 0.397 | 0.123 | 0.291 | 0.275 | 0.271 | 0.159 | 0.188 | 0.128 | 0.212 | 0.080 | 0.103 | 0.166 | 0.267 | - | 0.284 |
| Mean | 0.204 | 0.308 | 0.160 | 0.429 | 0.170 | 0.302 | 0.317 | 0.306 | 0.195 | 0.224 | 0.175 | 0.234 | 0.179 | 0.157 | 0.188 | 0.283 | 0.202 | - |
