# Supplementary material for: Genetic structure, relationships and admixture with wild relatives in native pig breeds from Iberia and its islands
Source: Genet Sel Evol. 2013 Jun 14;45(1):18. doi: 10.1186/1297-9686-45-18 (PMC3698160; doi:10.1186/1297-9686-45-18)
Supplement: Additional file 2: Figure S1 — Geographic distribution of the domestic pig breeds studied. Map showing spread of the 15 domestic breeds analysed. Figure S2. Estimated posterior probabilities of the data for different number of inferred clusters in the analysis with Structure. Likelihood of different number of ancestral populations given the observed breed genetic diversity. [file 1297-9686-45-18-S2.docx]

Figure S1. Geographic distribution of the domestic pig breeds studied [Alentejano (ALE), Bísaro (BIS) and Malhado de Alcobaça (MAL) from Portugal; Celta (CEL), Chato Murciano (CHM), Entrepelado (ENT), Euskal Txerria (ETX), Lampiño (LAM), Manchado de Jabugo (MJA), Negro Canario (NCA), Negro de Formentera (NFO), Negro de los Pedroches (NPE), Negro Mallorquín (NMA), Retinto (RET) and Torbiscal (TOR)].


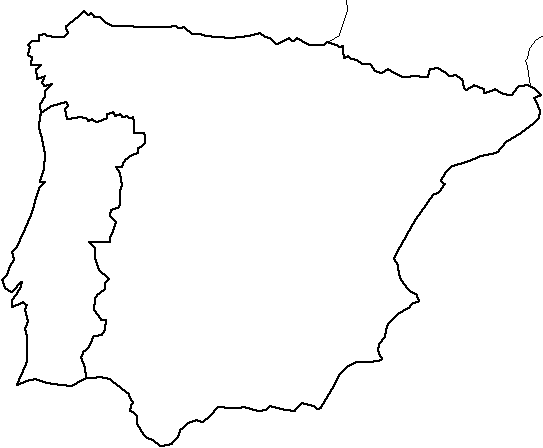

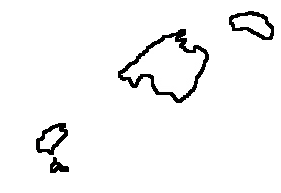


ALE

MAL

BIS

CEL

ETX

CHM

NCA

NFO

NMA

NPE

RET

TOR

ENT

LAM

MAJ

Figure S2. Plot of estimated posterior probabilities of the data [Ln Pr(X|K)] for number of inferred clusters ranging from K = 2 to K = 17, with the mean probability for 10 runs (⭘) and the corresponding standard deviation represented by a vertical bar.


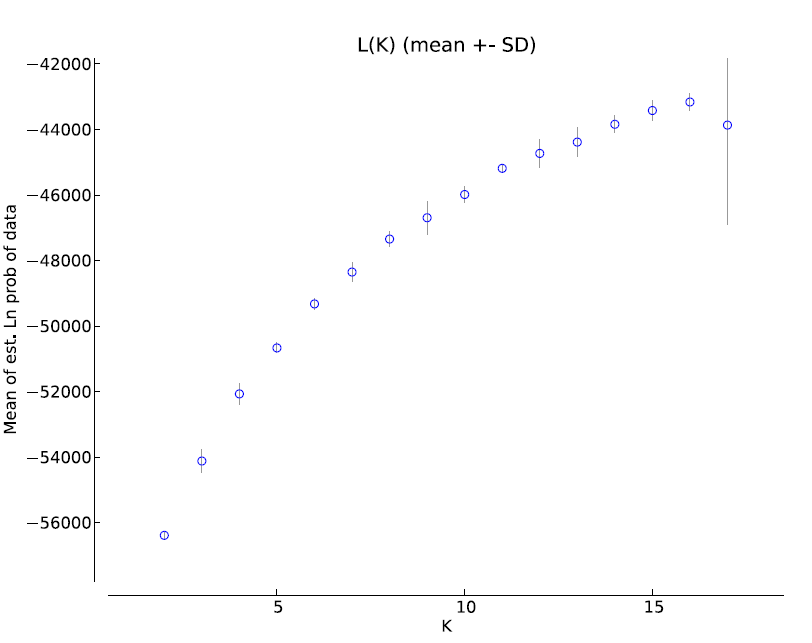


Ln Pr(X|K)]
